# A molecular dynamics study of adenylyl cyclase: The impact of ATP and G-protein binding

**DOI:** 10.1371/journal.pone.0196207

**Published:** 2018-04-25

**Authors:** Elisa Frezza, Juliette Martin, Richard Lavery

**Affiliations:** MMSB, Univ. Lyon I / CNRS UMR 5086, Institut de Biologie et Chimie des Protéines, Lyon, France; UMR-S1134, INSERM, Université Paris Diderot, INTS, FRANCE

## Abstract

Adenylyl cyclases (ACs) catalyze the biosynthesis of cyclic adenosine monophosphate (cAMP) from adenosine triphosphate (ATP) and play an important role in many signal transduction pathways. The enzymatic activity of ACs is carefully controlled by a variety of molecules, including G-protein subunits that can both stimulate and inhibit cAMP production. Using homology models developed from existing structural data, we have carried out all-atom, microsecond-scale molecular dynamics simulations on the AC5 isoform of adenylyl cyclase and on its complexes with ATP and with the stimulatory G-protein subunit Gsα. The results show that both ATP and Gsα binding have significant effects on the structure and flexibility of adenylyl cyclase. New data on ATP bound to AC5 in the absence of Gsα notably help to explain how Gsα binding enhances enzyme activity and could aid product release. Simulations also suggest a possible coupling between ATP binding and interactions with the inhibitory G-protein subunit Gαi.

## Introduction

Cyclic adenosine monophosphate (cAMP) is a universal second messenger in signal transduction based on G-protein coupled receptors (GPCR) in eukaryotes [1]. It is responsible for amplifying stimuli received by the cell [2–6]. It binds and regulates kinases and ion channels, whose activity subsequently determines how the cell will respond to the stimuli [7,8].

**Fig 1:**
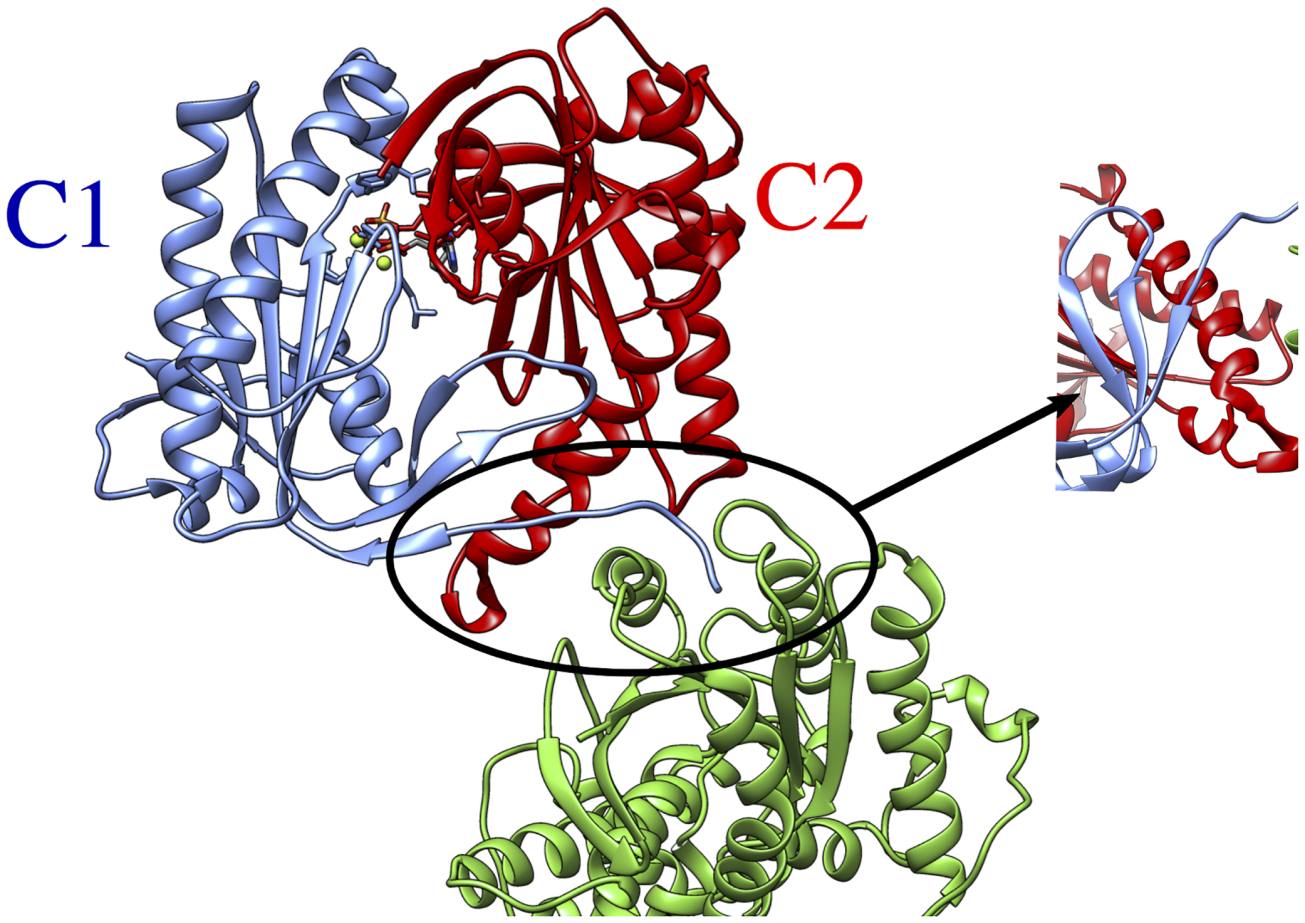
Structure of the cytoplasmic segment of the AC5 isoform of adenylyl cyclase in complex with ATP and the activating G-protein Gsα viewed from the side closest to the cell membrane. The C1 and C2 subunits of AC5 are colored blue and red respectively and Gsα is colored green. ATP is shown in a CPK representation with standard chemical coloring. A close-up view of the helix-helix interactions between AC5 and Gsα is provided (circle).

It is consequently not surprising that cAMP levels must be tightly controlled and the enzymes responsible for cAMP synthesis are indeed highly regulated [9]. This family of enzymes, the adenylyl cyclases (also commonly known as adenylate cyclases), has nine members (hereafter termed AC1-9). Each member of the family has specific regulatory properties and tissue distributions [10,11], however they all convert adenosine triphosphate (ATP) into cAMP via a cyclization reaction. Eukaryotic ACs share a similar topology with a variable N-terminus (NT) and two repeats of a membrane-spanning region followed by a cytoplasmic region [12,13]. The latter is divided into two pseudosymmetric domains, termed C1 and C2, each containing approximately 230 amino acid residues and sharing roughly 40% sequence identity. The N-terminal and C-terminal portions of C1 and C2 are the most variable regions among the different AC isoforms and among different organisms (see Fig 1).

The catalytic site of ACs is located at the C1/C2 interface and binds a molecule of ATP accompanied by two magnesium ions. The adenine moiety of ATP forms hydrophobic interactions in the cleft between the C1 and C2 domains and its N1 and N6 sites form H-bonds with Lys 938 and Arg 1018 deep in the active site cavity (the residue numbering used here is from the chimeric rat/dog X-ray structure of AC5, PDB entry: 1CJK [14]). The triphosphate moiety of ATP forms extensive H-bond interactions with residues of both the C1 (Thr 401, Phe 400, Gly 399, Arg 484) and C2 domains (Lys 1065, Arg 1029). The two magnesium ions within the active site are octahedrally coordinated by the O3’ oxygen of the ribofuranose alcohol, the triphosphate group, and the residues Asp 396, Asp 440 and Ile 397 of the C2 domain. Despite this ensemble of interactions, the triphosphate-Mg complex remains exposed to the solvent and interacts with several water molecules (see Fig 1).

Upon GPCR activation [15–17], the stimulatory G-protein subunit alpha (Gsα) is released from its cognate receptor and binds to and activates the AC enzyme via the subunit’s interaction with the C2 domain [11,15,18,19]. A number of other AC modulators, either stimulators or inhibitors of cAMP synthesis, are known. These include the inhibitory G-protein subunits Gαi and Gβγ, calcium ions, calmodulin and a variety of kinases. This indicates that the AC enzymes are capable of integrating diverse signals in a subtle and specific manner [20–23].

Structural information on a single domain of the cytoplasmic catalytic core of AC [24] and on a complex containing both AC catalytic domains bound to an active conformation of the stimulating Gsα, with or without a bound ATP analog, is available [25]. However, the structure and function of the transmembrane regions, each predicted to contain six membrane-spanning helices, is unknown and we also lack structural data on the N- and C-terminal portions of the C1 and C2 domains. In both structures, AC was indeed co-crystallized with forskolin, a plant-derived activator of cAMP production by AC [26,27]. However, no mammalian forskolin analogues have been identified that could regulate ACs via the forskolin binding pocket [28,29]. Moreover, there is no data on the enzyme bound to ATP (or an ATP analog) in the absence of activating Gsα, so it is difficult to understand how the latter protein actually activates adenylyl cyclase.

In order to address the latter question, we have used molecular dynamics simulations to study the impact of Gsα and also ATP binding on the structure and flexibility of AC. It is worth stressing that the cytoplasmic domains of AC5, structurally characterized by X-ray crystallography, are capable of reproducing many of the regulatory properties of the wild type enzyme and therefore can be used as working models to investigate the regulation mechanisms of AC [30,31]. Since different AC isoforms also respond differently to the same stimuli, we have used homology modeling to be able to work with a single isoform from a single organism. We have chosen to study the mouse AC5 isoform. This isoform notably plays a key role in a variety of neuronal GPCRs-based signal cascades [21,32,33].

**Fig 2:**
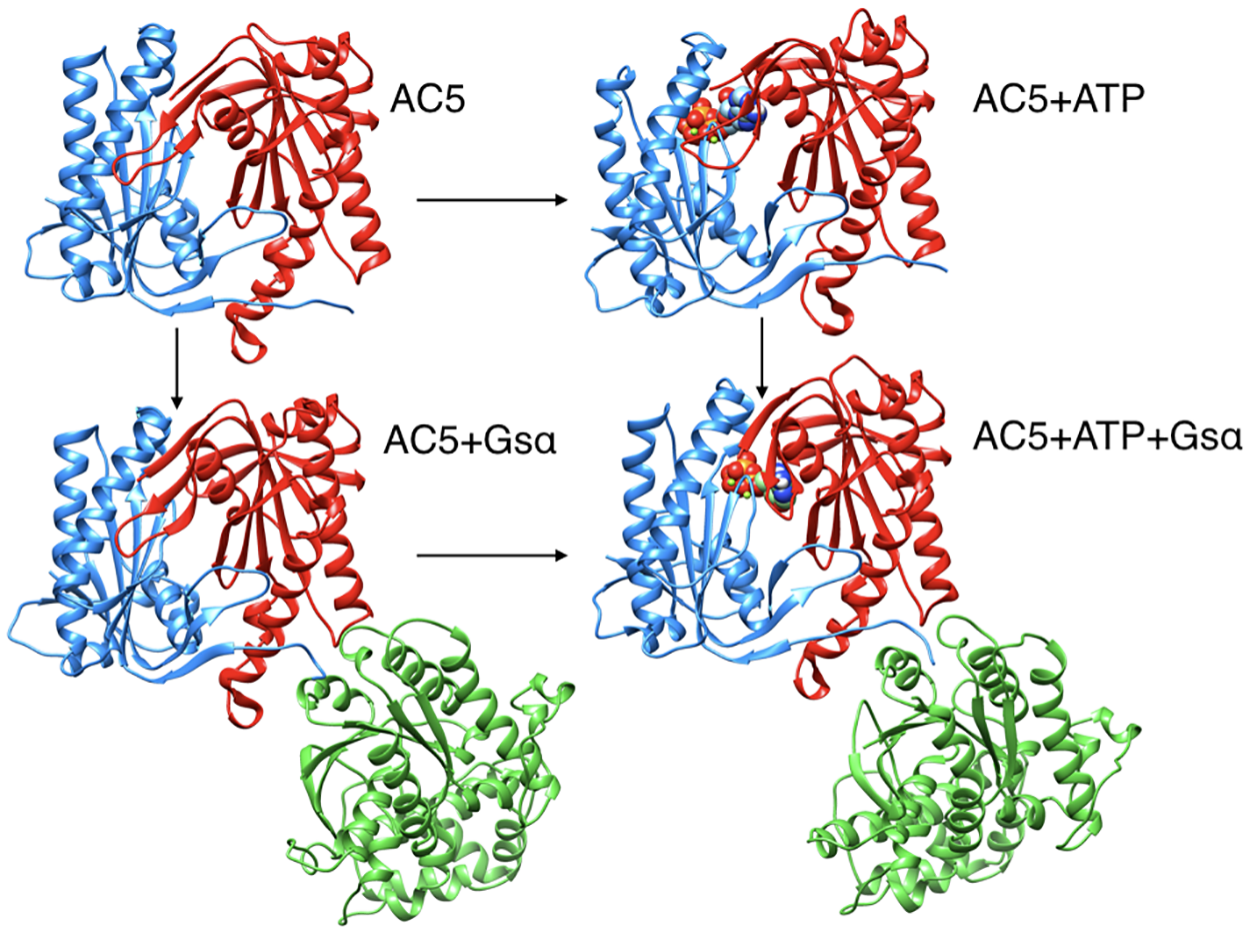
Structure of the cytoplasmic segment of the AC5 isoform of adenylyl cyclase and of its complexes with ATP and the activating G-protein Gsα viewed from the side closest to the cell membrane. Proteins are shown as backbone ribbons. The C1 and C2 subunits of AC5 are colored blue and red respectively and Gsα is colored green. ATP is shown in a CPK representation with standard chemical coloring. In each case, the structures are averages taken from the molecular dynamics simulations described in this work.

The all-atom microsecond-scale simulations of the AC5 and its complexes with ATP and/or Gsα studied here (Fig 2) help to explain how binding changes the properties of AC5 and notably to understand the stimulatory effect of Gsα. For comparison, we also carry out some studies of the impact of the plant stimulating agent forskolin (see below).

While we are interested in the stimulation of AC5 function, we remark that recent work from Van Keulen and Rothlisberger [34] provides information of the inhibition of AC5 function, by carrying out simulations on Gαi bound to a putative site within C1 (by analog with the Gsα site on C2). Note that, in contrast to Gsα, the active form of Gαi is myristoylated on its N-terminal [35–37] (see also the conclusions to this article).

## Materials and methods

### Starting structures

Homology models for the mouse sequences of AC5 with or without ATP (adenosine triphosphate) and/or Gsα (the α subunit of the guanosine nucleotide binding protein) were built as follows. We only take into account the cytoplasmic domains of AC5 (C1 and C2 in Fig 1) because no structural data exist on the linkers to the membrane. It has also been shown that the soluble domains of AC5 are enzymatically competent [30,31] and no impact of the membrane anchorage on the stimulation of AC5 has been found [38,39]. We also removed forskolin from the crystallographic structures, because no mammalian forskolin analogues have been yet identified that might regulate ACs [21]. Lastly, we did not include any modification to Gsα (like the majority of G protein α subunits, Gsα is not N-myristolated) [39,40].

The model of the cytoplasmic segment of mouse AC5 without ATP or Gsα was generated with Modeller v9.12 [41] using the PDB structure 1AZS (chimera AC5 rat/ AC2 dog) [25]. This protein has a 98% sequence identity with the domain C1 and a 57% identity with C2 and is therefore a reliable template. The model of mouse AC5 with ATP and magnesium ions was generated using the same protocol using 1CJK as template [14]. The model of the mouse Gsα protein in its bound form with Mg^2+^ and GTP (guanosine triphosphate) as ligands was also generated with Modeller starting from bovine Gsα (80% sequence identity with the mouse protein) again taken from 1CJK. In each case, 100 homology models were generated and the structure with the lowest DOPE score [42] was selected for the simulations. The starting conformations for the AC5+Gsα and the AC5+ATP+Gsα complexes were obtained by molecular docking starting from the homology models of isolated AC5 and of Gsα using the Cluspro webserver [43]. The resulting positions of Gsα were close to those observed in the 1AZS and 1CJK complexes (0.8–1.5 Å for the binding α-helix of Gsα and 2.3–2.6 Å for the full backbone), although some change in its position is to be expected given residue substitutions at the ends of the α-helices of the C2 domain that bind to the G-protein (introduced during the homology modeling used to develop a non-chimeric AC5 structure).

### Molecular dynamics simulations

Molecular dynamics simulations were performed with the GROMACS 5 package [44–48] using the Amber 99SB-ILDN force field for proteins that has been shown to yield an accurate description of many structural and dynamical properties of proteins [49–52]. Side chain protonation states of titratable amino acids were assigned using a value of pH = 7.4 with the help of the pdb2pqr software [53]. Capping acetyl and methyl-amino groups were added to the N and C termini of both AC5 domains and of Gsα. The four states we study (AC5, AC5+ATP, AC5+Gsα and AC5+ATP+Gsα) were each placed in a truncated octahedral box and solvated with TIP3P water molecules [54] to a depth of at least 11 Å. The solute was neutralized with potassium cations and then K^+^Cl^-^ ion pairs [55] were added to reach a physiological salt concentration of 0.15 M. Parameters for ATP and GTP were taken from [56]. The parameters for Mg^2+^ came from [57]. This new set of parameters was developed to improve the kinetic properties of Mg^2+^ ions with water and with the phosphate ion and it was implemented in Amber99. This new set of parameters also provided a better description of the structure of Mg^2+^-phosphate binding than previous sets (these interactions are naturally important in our simulations in the presence of ATP) [57]. Hence, the combination of Amber 99SB-ILDN and the new set of parameters of Mg^2+^ ions is currently the best choice to reproduce the dynamics of AC5 and Gsα, and to properly describe the interactions of Mg^2+^ with AC5 and ATP.

Long-range electrostatic interactions were treated using the particle mesh Ewald method [58,59] with a real-space cutoff of 10 Å. We used virtual interaction sites for the hydrogens and bond lengths were restrained using P-LINCS [47,60], allowing a time step of 4 fs [61]. Translational movement of the solute was removed every 1000 steps to avoid any kinetic energy build-up [62]. After energy minimization of the solvent and equilibration of the solvated system for 10 ns using a Berendsen thermostat (τ_T_ = 1 ps) and Berendsen pressure coupling (τ_P_ = 4 ps) [63], the simulations were carried out in an NTP ensemble at a temperature of 310 K and a pressure of 1 bar using a Bussi velocity-rescaling thermostat [64] (τ_T_ = 1 ps) and a Parrinello-Rahman barostat (τ_P_ = 1 ps) [65]. Simulations were carried out using typically between 72 and 120 computer cores depending on the system size, which allowed a production rate of about 100 ns/ day. Analysis was carried out on a 1.1 μs production segment for each simulation, following a 400 ns equilibration period.

### Molecular dynamics simulations in the presence of forskolin

Although no mammalian forskolin (FOK) analogues have been yet identified that might regulate ACs, FOK is known to stimulate cAMP production by adenylyl cyclase. We can therefore ask if forskolin and Gsα have similar conformational and dynamic impacts on AC5. To do this, we began with the central structure of the largest clusters obtained in the last 500 ns of the trajectories of AC5 and AC5+ATP and we placed the forskolin in the pocket identified crystallographically. For FOK, we used RESP charges [66] and Amber 99SB with GAFF for the topology parameters. Using the protocol described above for the molecular dynamics simulations, we performed 1000 ns of simulation for each system (AC5+FOK and AC5+ATP+FOK). Analysis was carried out on the final 600 ns production segment for each simulation.

### Analysis of the simulations

We analyzed our MD simulations using average structures, time series and average values of RMSD (root mean square deviation) and RMSF (root mean square fluctuations), specific geometrical measurements described below, protein-protein and protein-ligand interface characteristics and, in some cases, residue-by-residue conformational and dynamic properties.

The C1/C2 interface was characterized using three quantities: the gap volume, the change of accessible surface area upon binding (ΔASA), and the gap index [67,68]. The gap volume was calculated using the procedure developed by Laskowski [67], which estimates the volume enclosed by any two molecules. The change of solvent accessible surface area on complexation is defined as:

$$\Delta \text{ASA}=\frac{\left({\text{ASA}}_{A}+{\text{ASA}}_{B}-{\text{ASA}}_{AB}\right)}{2}$$ (1)

where A and B represent the monomeric states, AB is the dimeric state. The solvent accessible surface area was calculated by using the same radii as the gap volume calculation [67]. The gap index evaluates the complementarity of the interacting surfaces in a protein-protein complex and is defined as Gap index = Gap volume / ΔASA. Typical values of the gap index range from 1 → 5. Lower values characterize interfaces with better structural complementarity. In general, protein homodimers have significantly smaller values than heterodimers [68].

**Fig 3:**
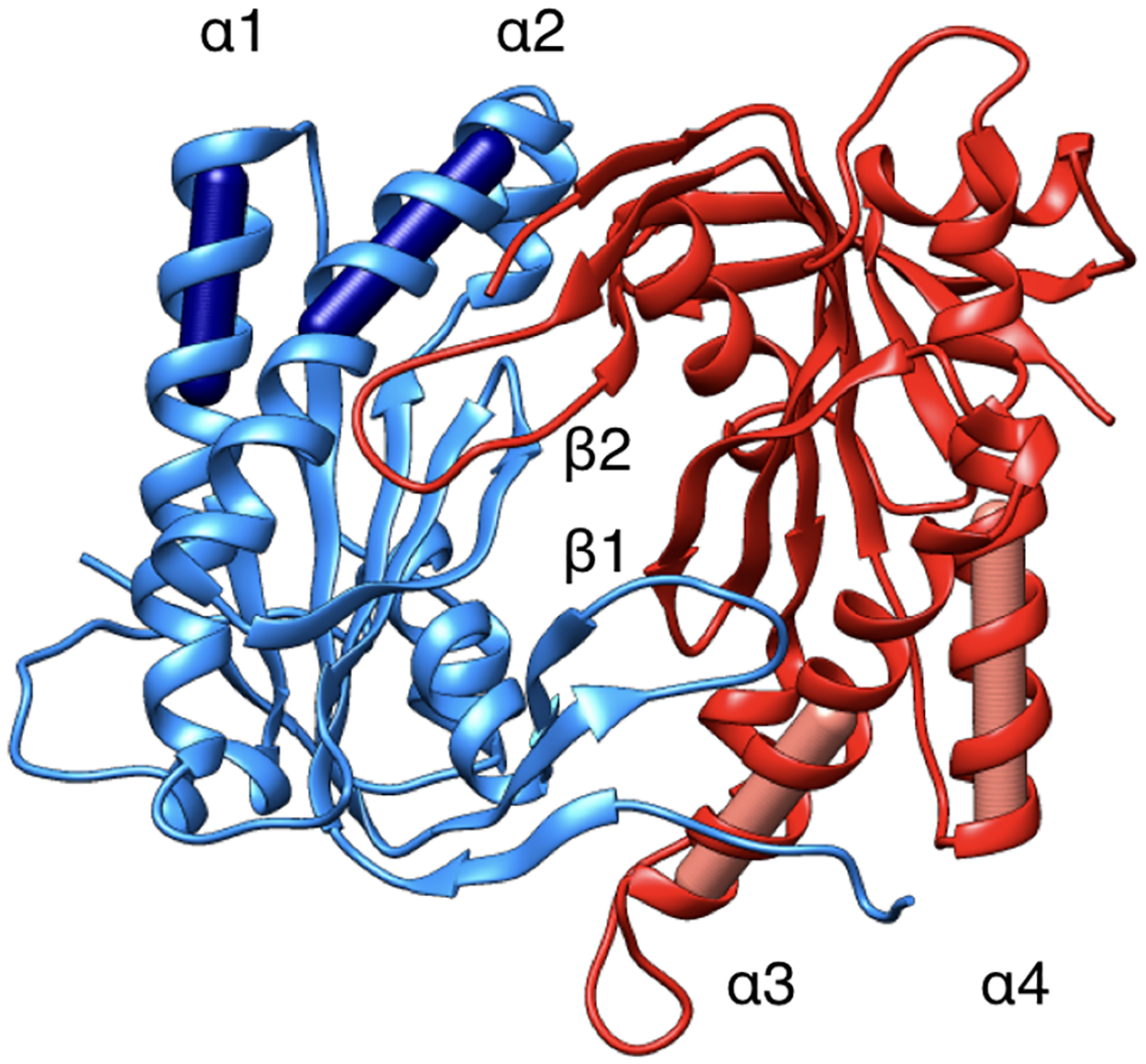
Illustration of the α-helices involved in binding the stimulatory protein Gsα (α3 and α4) and potentially binding the inhibitory protein Gαi (α1 and α2). The cylinders represent the helical axes used in determining the angles between the pairs of helices. The C1 and C2 domains are colored blue and red respectively. Two relevant β-loops (β1 in domain C1:494–503 and β2 in domain C2:1010–1016) are also indicated.

In order to characterize potential G protein binding sites we computed the angle α_C2_ between the pairs of α-helices within domain C2 that bind Gsα (termed α_3_ and α_4_ in Fig 3) and also the angle α_C1_ between the quasi-symmetric pair of helices within domain C1 (termed α_1_ and α_2_ in Fig 3). The latter helices may constitute a binding site for the inhibitory G-protein subunit Gαi under the hypothesis of Gαi binding in a symmetrical configuration [36]. The angles were measured using helical axes derived from the residues that remain in stable α-helical conformations throughout the simulations (C1: 408–420 and 468–475, C2: 910–918 and 978–988, see Fig 3).

In order to address the collective motions involved in the stimulation pathway, we carried out a principal component analysis (PCA) on the all-atom molecular dynamics simulations. We considered only the backbone heavy atoms (C, Cα and N) and computed a least squares fit on the molecular system and the mass-weight covariance matrix using the GROMACS package [48]. In order to analyze the motions corresponding to individual eigenvectors, we filtered the original trajectory and projected out the part along the selected eigenvector. We then analyzed the movements associated with the first five eigenvectors that contribute most to the global movements of the system.

## Results

In the absence of any structural information on a single non-chimeric isoform of adenylyl cyclase, or on the catalytic domains of the enzyme without the stimulating G-protein subunit Gsα, we use a combination of homology modeling and molecular dynamics to try to understand how protein or ligand binding can modify the conformation or the dynamics of AC5 and subsequently impact on its enzymatic function.

We have studied the behavior of four molecular species (see Fig 2): isolated AC5, AC5 with ATP and two Mg^2+^ ions in its active site (AC5+ATP), AC5 bound to the activating G-protein subunit Gsα (AC5+Gsα+GTP) and AC5 bound to both ATP and Gsα (AC5+ATP+Gsα+GTP). For each of these species, we generated 1.5 μs MD trajectories in an aqueous environment with a physiological salt concentration (0.15 M KCl). The first 400 ns of each trajectory were treated as equilibration of the system and analysis was carried out only on the remaining 1.1 μs. Data shown in S1–S3 Tables confirm the stability of the simulations during the segment used for analysis.

**Fig 4:**
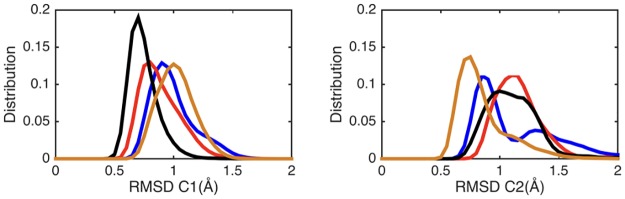
Probability distributions of RMSD for C1 (left) and C2 domains (right) calculated along the MD trajectories with respect to the corresponding average structure: AC5 (blue), AC5+ATP (red), AC5+Gsα (ochre) and AC5+ATP+Gsα (black).

We begin by considering the global impact of ATP or Gsα binding on AC5. RMSD calculations with respect to the average MD structure of each AC5 domain show that the isolated protein (blue lines in Fig 4) is moderately flexible. Most of the flexibility in domain C1 concerns the unstructured N-terminal. However, as shown in Fig 4, domain C2 visits three conformational substates that involve large movements of loop β2 (see Fig 3), changing its distance from the unoccupied ATP binding site.

**Fig 5:**
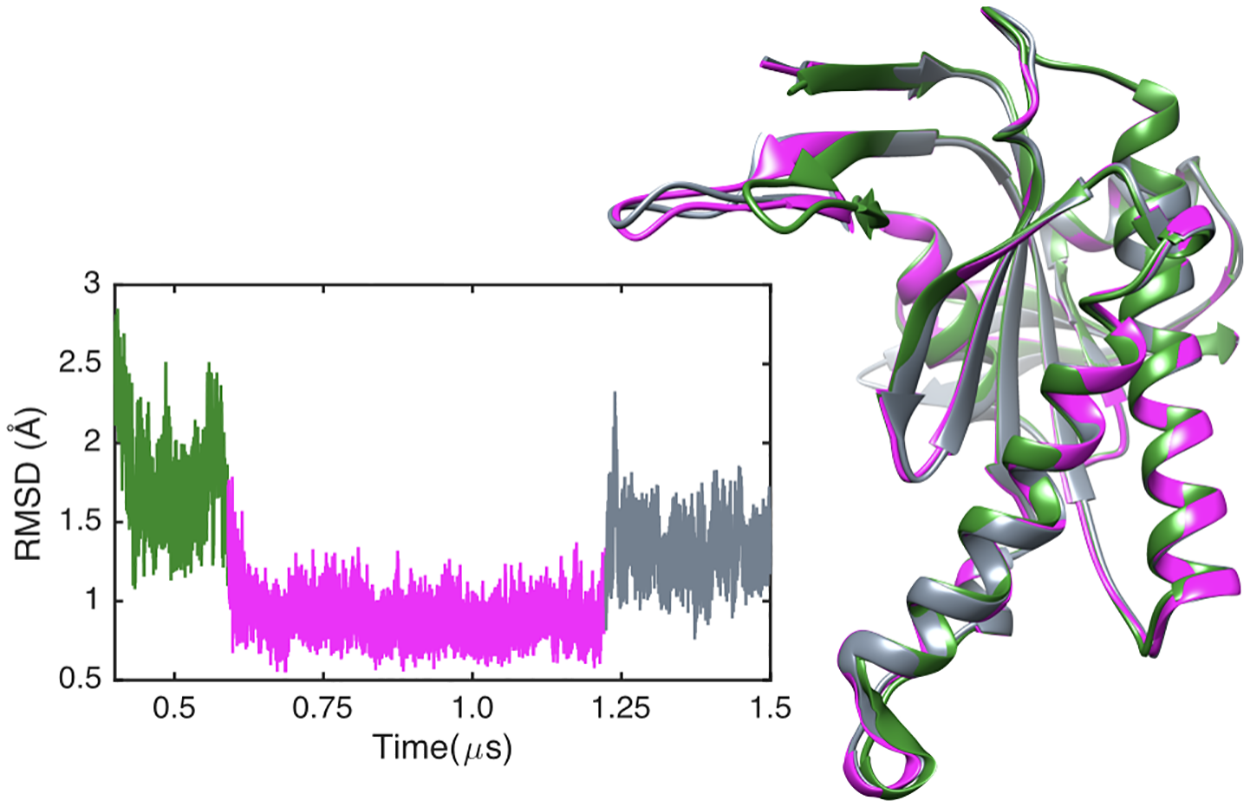
Left: RMSD time series of domain C2 of AC5. Right: Three average substate structures of C2 (green, magenta and grey) show that changes mainly involve the β2 loop.

Adding ATP to the AC5 binding site has a significant effect on both the structure and the dynamics of the protein. Although the ATP binding site involves both domains of AC5, ATP has stronger interactions with domain C1 via its associated Mg^2+^ ions, notably with residues Asp 396 and Asp 440. It is thus not surprising that ATP binding slightly rigidifies domain C1 (red lines in Fig 4). However, it also leads to an increased overall flexibility in domain C2, coupled with the selection of a single substate for the β2 loop (the longest-lived substate in isolated AC5, see Fig 5). ATP remains mobile and internally flexible within its binding site with the adenine base regularly changing its orientation with respect to the triphosphate tail. Although interactions between the terminal phosphate group of ATP and Lys 1065 (belonging to loop β2) are maintained, interactions between the penultimate phosphate and Arg 1029, a key functional residue, are absent, the arginine side chain being separated from its target oxygen atom by roughly 11 Å.

ATP binding also turns out to have more global effects on AC5. First, the angles between the pairs of α-helices in both AC5 domains are modified: significantly increased in domain C1 (by 18°, see supplementary S1 Table) and reduced in C2 (by 7°), involving a global movement of helix α3 (see Fig 5). We also see an overall tightening of the C1/C2 interface, with a decrease in the average gap index from 3.1 Å for isolated AC5 to 2.8 Å once ATP is bound. In terms of flexibility, ATP binding mainly rigidifies the binding site region of AC5, although it also increases the flexibility of the C-terminals of helices α1 and α3 and the N-terminal of α4.

**Fig 6:**
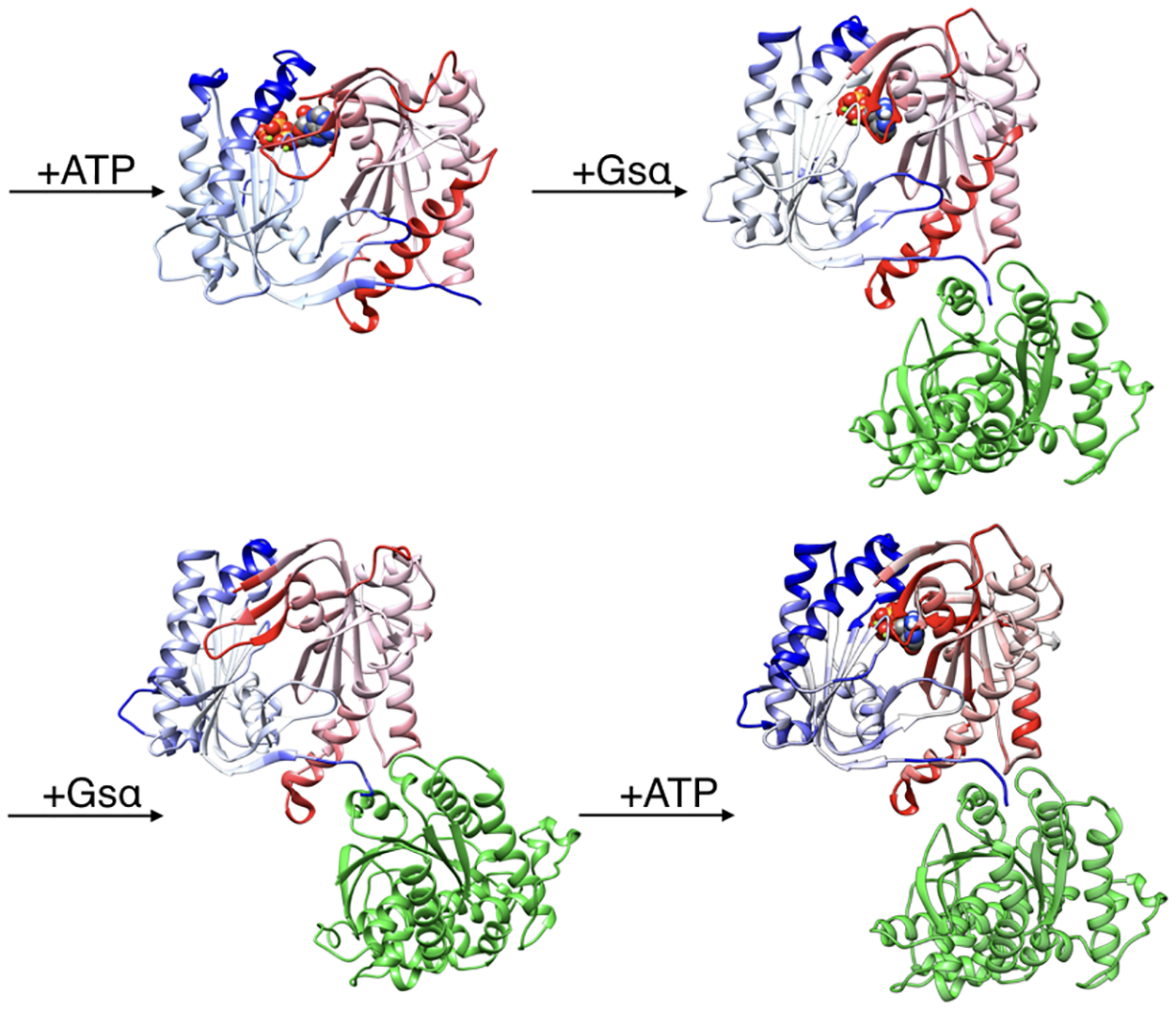
Changes in the conformation induced by ATP and Gsα binding. More intense colors (blue for domain C1 and red for domain C2) correspond to larger movements compared to the preceding structure (i.e. AC5 and AC5+ATP for the top line and AC5 and AC5+Gsα for the bottom line) on a scale of 0 → 4 Å.

**Fig 7:**
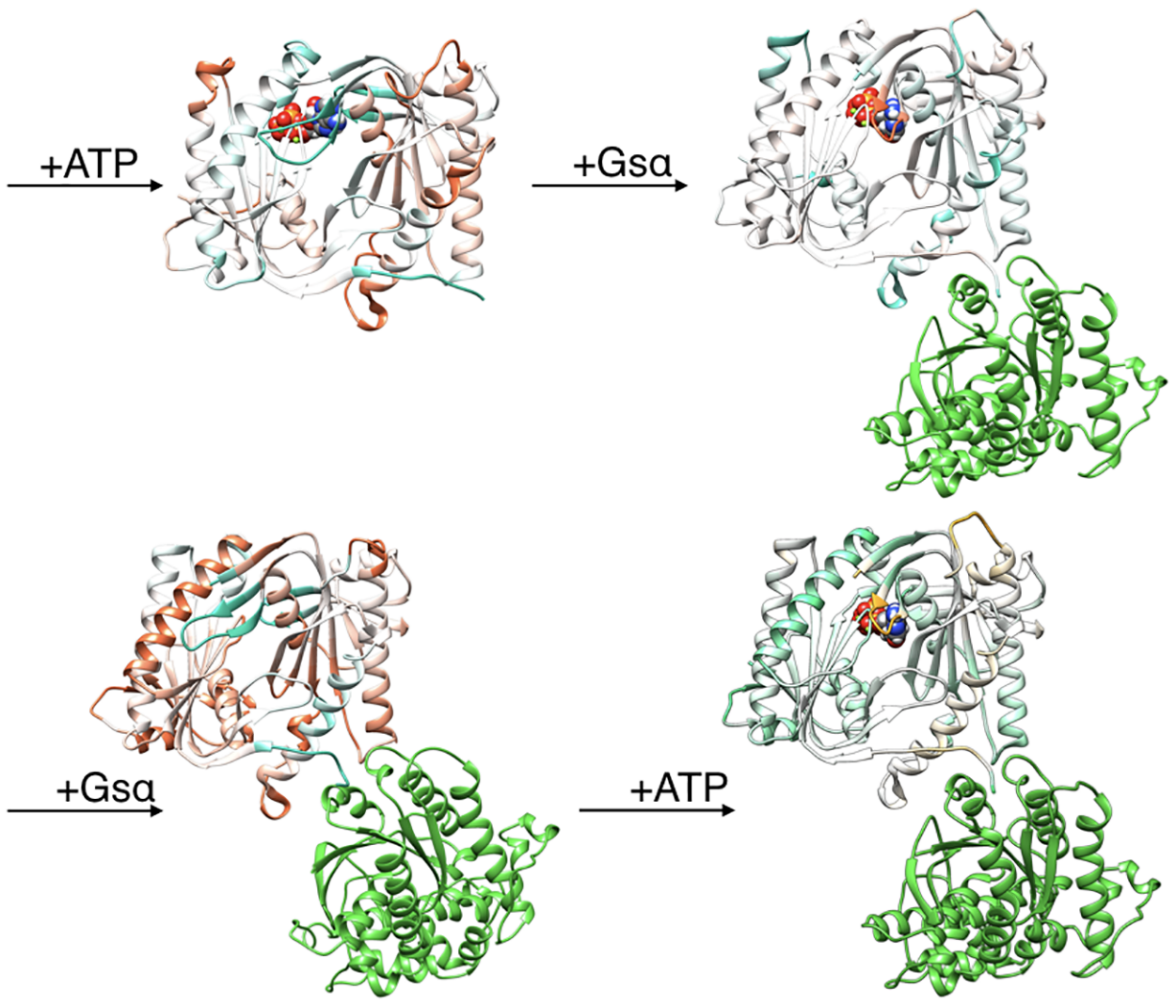
Changes in RMSF induced by ATP and Gsα binding. More intense colors (orange for increased flexibility and cyan for decreased flexibility) correspond to differences with respect to the preceding structure (i.e. AC5 and AC5+ATP for the top line and AC5 and ACT+Gsα for the bottom line) on a scale of -1.2 Å → +1.2 Å.

If we now add Gsα to the AC5+ATP complex we see a further movement of helix α3 (see Fig 6), although this has little effect on the angle α_C2_ where the G-protein binds (see S1 Table). In the more distant C1 domain, the angle α_C1_ is also only slightly affected despite some movement in the helices α1 and α2. The most striking effects of Gsα binding involve the ATP active site, where a β-sheet movement brings the key residue Arg 1029 back into its active location binding the primary phosphate group of ATP and globally rigidifies the binding site (with a concomitant effect on domain C1, see Figs 4 and 7). Simultaneously, we see an opening of loop β2 away from the active site, re-adopting the first substate seen in isolated AC5 and increasing its flexibility (see Fig 7). This change is coupled to a looser C1/C2 interface whose gap index increases from 2.8 to 3.8, putting it in the realm of non-obligate protein-protein interfaces [67,68].

As well as modifying the ATP binding site, Gsα also modifies the mobility of ATP whose average RMSD decreases by 50% from 0.6 Å to 0.3 Å (see S3 Table and S1 Fig). In this state, ATP has strong interactions with both domains. As well as stable binding of the triphosphate tail to the key residues Arg 1029 and Lys 1065, there are also stable interactions of Asp 460 and Asp 396 with the two Mg^2+^ ions. This leads to a 25% reduction on the exposed surface of ATP, mostly due to stronger interactions with domain C2 (see S3 Table).

At this stage, it is interesting to compare the impact of Gsα with that of the stimulating agent forskolin (FOK). We find that, like Gsα, FOK binding also induces a change in the helix α3 (see S3 and S4 Figs), that has an effect on the angle α_C2_ where the G-protein binds (see S2 Table). The change of this angle depends on the presence or absence of the ATP. FOK binding also induces changes at the ATP active site in a similar fashion to Gsα: the movement of the α-helices at the interface brings the key residue Arg 1029 back into its active location and globally rigidifies the binding site. However, in the case of AC5+ATP+FOK, there is a slight rotation of the two domains and a movement of the helices at the interface that are coupled to a stronger C1/C2 interface and a small reduction of the gap index. Most interestingly, FOK binding reduces ATP mobility and establishes stable interactions with the key residues for cAMP production. These changes lead to a 10% reduction of the exposed surface of ATP, mostly due to stronger interactions with domain C2 (see S3 Table and S3 and S4 Figs).

Although it seems probable that ATP is already bound to AC5 before its enzymatic action is activated by Gsα binding, we have also considered the alternative scenario where Gsα binds prior to ATP. This involved simulating the AC5+Gsα complex shown in Fig 2. As seen in Figs 6 and 7, Gsα binding to isolated AC5 has a similar effect to its binding to the AC5+ATP complex, with two significant differences. First, the loop β2 is rigidified and does not move away from the (empty) ATP binding site. However, the overall flexibility of domain C1 is increased by the lability of helix α2. Second, the C1/C2 interface remains as tight as in isolated AC5.

A further interesting observation is that the ATP/Gsα interface is considerably less stable when the G-protein is bound to AC5 alone than when ATP occupies its AC5 binding site. As shown in S3 Table, ATP decreases the AC5/Gsα gap index from 3.2 to 2.7, a value close to that of the AC5 C1/C2 interface in the presence of ATP.

## Discussion

The first observation that can be made from these microsecond-scale simulations is that strong allosteric coupling exists within AC5. Thus, ATP binding modifies not only the position of the proximal α-helices of domain C1, but also those at the distal end of domain C2. It also significantly improves the steric complementarity of the C1/C2 interface. Subsequent Gsα binding to domain C2 modifies the pair of α-helices belonging to distal end of domain C1, weakens the C1/C2 interface, locks ATP into its active conformation by completing bonds with all the key AC5 residues and also displaces and renders flexible the β2 loop (see S2 Fig). Equally, the AC5-Gsα interface is significantly stabilized by the presence of ATP in its AC5 binding site. All of these changes involve coupling through AC5 over distances of tens of angstroms. The mechanism behind this coupling can be better understood with the help of a principal component analysis of the corresponding trajectories. The results are illustrated in four videos included in the supplementary data to this article. By comparing the low frequency modes of AC5 alone (S1 Video) with AC5:ATP (S2 Video), one can see that ATP binding improves the C1/C2 domain interface, opens the α1/α2 angle, the presumed Gαi binding site, and closes the α3/α4 angle at the Gsα binding site. As observed in the RMSF profile, the β-sheet β2 is no longer mobile in the presence of ATP. In contrast, in the AC5:ATP:Gsα complex (S4 Video), as in apoAC5, β2 is mobile and its movements are strongly linked to movements of the α1-α4 helices. Communication between the Gsα binding site in domain C2 and the postulated Gαi site in domain C1, through the domain interface, particularly striking in the AC5:Gsα complex (S3 Video), but is in fact observed in all four trajectories studied here, although the relative movements of the two domains change in each case.

Turning now to the enzymatic function of AC5, it is known that a hydrogen bond between the highly conserved Arg 1029 residue and the primary phosphate group of ATP play an important role on the production of cAMP from ATP and substitution of arginine by alanine at this position leads to a 100-fold reduction in enzymatic activity [69]. Hybrid QM/MM free energy calculations have also stressed the role of this interaction and show a significant increase in the free energy barrier for the reaction when the same substitution is made [70].

**Fig 8:**
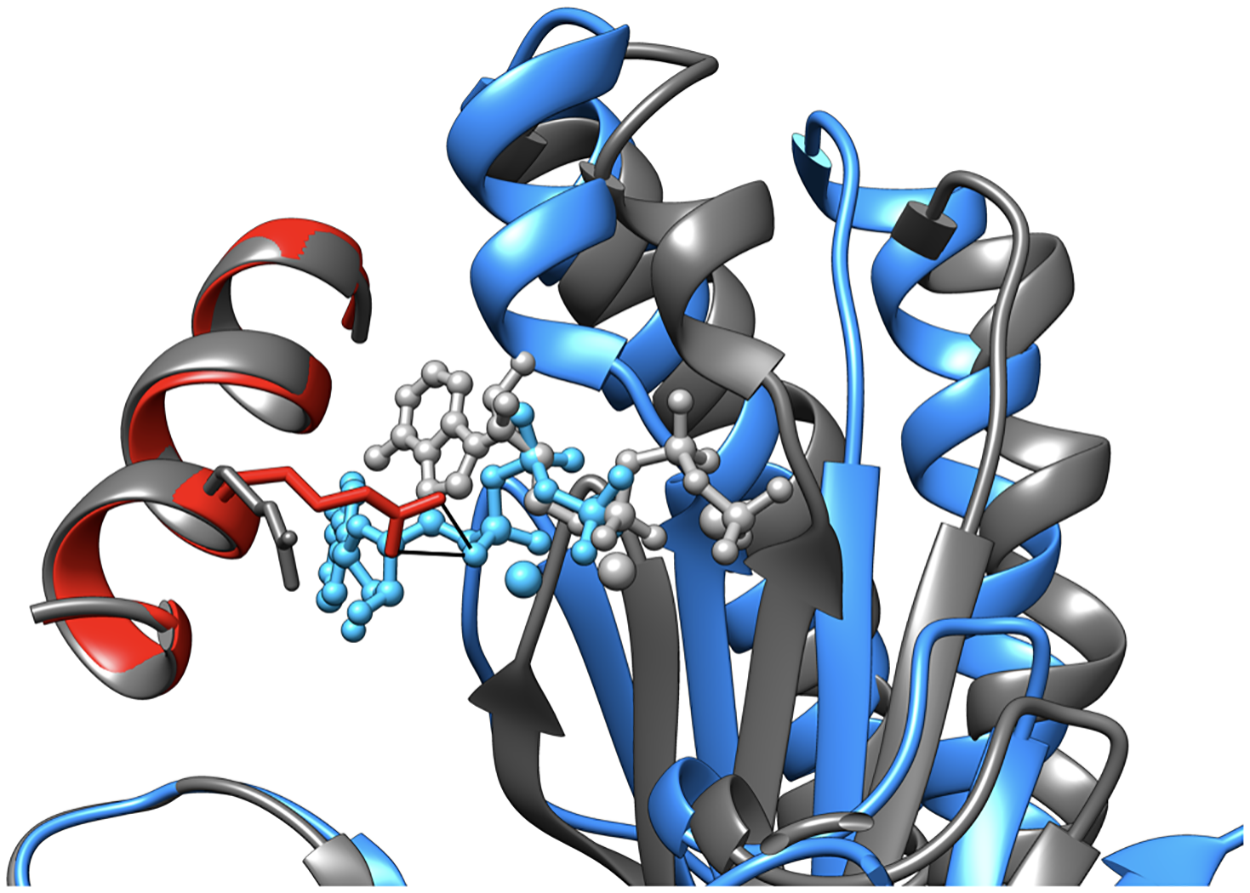
Movement of the β-sheet of AC5 domain C1 and ATP as a function of Gsα binding. The average conformation AC5 and ATP bound to Gsα is shown in blue (domain C1), red (α-helix of domain C2 carrying residue Arg 1029) and sky blue (ATP). The same elements of the average conformation without Gsα are shown in grey. The two complexes are superposed on the C2 domain of AC5. In the absence of Gsα, the β-sheet of domain C1 is shifted toward the viewer, breaking the interaction between ATP with Arg 1029 (black lines).

The present simulations show that this interaction is not formed unless Gsα is bound to AC5, helping to explain the stimulatory mechanism of Gsα. The presence of the Arg 1029-ATP interaction in the AC5+ATP+Gsα complexes reproduces the interaction seen in the chimeric crystal structure of adenylyl cyclase with an ATP analog, 1CJK [14]. Fig 8 shows why this interaction is absent in the simulated AC5+ATP complex. As mentioned above, there are significant differences in the overall structure of AC5 depending on the presence or absence of Gsα. These include a displacement of the β-sheet that carries the aspartic acid residues bound to ATP through the two chelated magnesium ions. In the absence of Gsα, the β-sheet movement thus also displaces ATP, placing the triphosphate tail too far away from the Arg 1029 side chain for an interaction to occur.

As already noted, FOK is a plant-derived agent known to stimulate cAMP production by adenylyl cyclase [26,27]. It is interesting to remark that our simulations show the Arg 1029-ATP interaction generated by Gsα binding is also recovered with FOK binding. Moreover, when FOK is bound to AC5, the primary phosphate group of ATP is more exposed to the solvent, thus suggesting the possibility to escape the products again similarly to our findings with Gsα. The only major difference with FOK involves the C1/C2 interface that become slightly stronger, in line with experimental observations [28,29], whereas it is weakened by Gsα.

Lastly, following earlier work that suggested that decreasing the mobility of ATP within the enzyme active site would enhance catalytic efficiency [71], we recall that Gsα not only modifies the position of ATP, but also reduces its positional and internal mobility within the AC5 binding site and this is another factor helping to explain its stimulatory effect. Once again, FOK binding induces similar effect on ATP and its binding pocket.

A second possible role for Gsα is linked to changes in the AC5 C1/C2 domain interface. While firmly blocking ATP in its binding site, Gsα concurrently weakens the AC5 interface, changing its average gap index from 2.8 Å to 3.8 Å, a value in the range of weak non-obligate protein-protein interfaces [68]. This change, coupled with the displacement and the increase in flexibility of loop β2 (S2 Fig) that is close to the ATP binding site could conceivably facilitate the escape of the products from the ATP binding site once the enzyme reaction has occurred.

In common with Gsα binding, ATP entering the AC5 binding site also impacts the conformation of the protein. The most interesting change is the opening up of the angle between the pair of α-helices of domain C1 (α_C1_). ATP causes this angle to increase on average from 26° to 44°, a value close to that of the equivalent helix pair in domain C2 where Gsα binds. The α_C1_ angle remains open when Gsα binds, but is not opened by Gsα binding alone (see S1 Table). It is not yet clear where the inhibitory G-protein Gαi binds to AC5, or how it acts. Given its structural similarity to Gsα, it could bind to domain C2 in the place of Gsα, but it could also bind to the pseudo-symmetric site of domain C1—if the angle between the corresponding α-helices α_C1_ was sufficiently large to accommodate the protein. We can at least conclude that if domain C1 is involved in Gαi inhibitor binding, then this would be less likely to occur until an ATP molecule enters the AC5 active site (and whether Gsα is already bound to domain C2 or not).

### Conclusions

We use molecular dynamics simulations in an attempt to better understand the behaviour of adenylyl cyclase, a key enzymatic player in cellular signalling cascades. Microsecond-scale simulations of the impact of binding ATP and/or the G-protein subunit Gsα to adenylyl cyclase help to explain features of this important signal transmission protein that are not easily derived from known experimental structures or from biochemical investigations of enzyme function. They notably provide information on a single, non-chimeric adenylyl cyclase isoform, AC5, and on the enzyme alone or bound to ATP, but in the absence of a stimulatory G-protein.

The simulations show that both ligand and protein binding create significant changes in structure, and in flexibility, throughout AC5 and due to a strong allosteric coupling existing within AC5, that is confirmed by a PCA analysis. They provide data that help to explain the stimulatory action of Gsα, whose binding quenches the conformational and positional fluctuations of ATP in the active site of AC5 and leads to the formation of a key Arg 1029-ATP interaction.

Our results also show that ATP binding at the AC5 domain interface results in a shift in flexibility from C1 to C2 (although the most flexible substates of C2 are eliminated), strengthens the C1/C2 interface and significantly opens the angle between the C1 α-helices than can potentially bind Gαi. Gsα binding to C2 has a lesser impact and the C1/C2 interface is unaffected. Lastly, simultaneous binding of ATP and Gsα resembles the state where ATP is bound alone, except that the C1/C2 interface is notably weakened and the C1 α-helices are somewhat less opened.

Although Gsα alone has a relatively small impact on AC5 dynamics it has a major effect when ATP binds, by limiting the conformational freedom of the bound ligand, correctly configuring it within its binding site and establishing key interactions between ATP and AC5. As discussed above, this change also significantly weakens the C1/C2 interface, opening the two domains at the ATP binding site and potentially facilitating the escape of the enzymatic reaction products from the ATP binding site. Similar results were also found when forskolin, a plant-derived agent known to stimulate cAMP production by adenylyl cyclase, is bound to AC5 (with the exception of the C1/C2 interface which remains strong, in line with experimental data). The similar changes induced within the ATP site by two very different molecular species argue in favor of their importance in the functional mechanism of adenylyl cyclase.

Our simulations also show that ATP binding could influence the binding of the inhibitory G-protein subunit Gαi, if the potential binding site within domain C1 (pseudo-symmetric with the domain C2 site used by Gsα) were to be involved. This work may be juxtaposed with the recent simulation study of AC5 inhibition by Gαi made by Van Keulen and Rothisberger [34]. By binding Gαi to a postulated C1 binding site, they find structural modifications that would disfavor both ATP and Gsα. Given the results presented here, it would be interesting to see what impact Gαi would have if ATP were already bound in its AC5 pocket and also whether Gsα and Gαi could nevertheless bind simultaneously to AC5. We are continuing our studies in this direction. Overall the results presented here stress the importance of obtaining structural data on adenylyl cyclase in the absence of stimulatory factors (forskolin or Gsα), and also of obtaining information on the impact of these same factors on dynamics of bound ATP (or rather of a close, non-reactive analogue).

## Supporting information

S1 Table Average and standard deviation of RMSD for the C1 and C2 domains of AC5, α-helix angles for both domains and characterization of the C1/C2 interface: Gap volume, change in accessible surface area (ΔASA) and gap index, for the systems studied with molecular dynamics simulation. To compute the RMSD we used the average structure as the reference state.
(PDF)

S2 Table Average and standard deviation of RMSD for the C1 and C2 domains of AC5, α-helix angles for both domains and characterization of the C1/C2 interface: Gap volume, change in accessible surface area (ΔASA) and gap index, for the systems studied with molecular dynamics simulation. To compute the RMSD we used the average structure as the reference state.
(PDF)

S3 Table Average and standard deviation of RMSD, ASA and ΔASA with domains C1 and C2 for ATP bound to AC5 in the absence or in the presence of Gsα and in the presence of FOK. (PDF)

S4 Table Average and standard deviation of the gap volume, ΔASA and the gap index of the AC5/Gsα interface in presence and in the absence of ATP. (PDF)

S1 Fig Change in mobility of ATP due to Gsα binding. Top: AC5+ATP, Bottom: AC5+ATP+Gsα. In both cases snapshots from the MD trajectories are superposed on the average structure of domain C1 of AC5 (blue). ATP is shown with standard chemical coloring.
(TIFF)

S2 Fig Change in position and flexibility of the β2 loop as a result of Gsα binding. The average structure of the AC5+ATP complex is shown in dark grey (ATP not shown). The same structure after Gsα binding is shown with coloring representing the change in RMSF (the cyan-white-orange variation covering variations of -1.2 Å to + 1.2 Å).
(TIFF)

S3 Fig Changes in the conformation induced by FOK on AC5 and AC5+ATP. More intense colors (blue for domain C1 and red for domain C2) correspond to larger movements compared to the preceding structure on a scale of 0 → 4 Å.
(PNG)

S4 Fig Changes in RMSF induced by FOK on AC5 and AC5+ATP. More intense colors (orange for increased flexibility and cyan for decreased flexibility) correspond to differences with respect to the preceding structure on a scale of -1.2 Å → +1.2 Å.
(PNG)

S1 Video PCA on the MD simulation of AC5. Global movement obtained by PCA on the MD simulation of AC5 alone (mode 1). The beta-sheet β2 is highly flexible and its movement is coupled with the relative movement of the domain C1 (blue) and C2 (red). The opening of the angle between the helices α1 and α2 (top left) is coupled with the closing of the angle between the helices α3 and α4 (bottom right). The important regions are highlighted with orange arrows.
(MOV)

S2 Video PCA on the MD simulation of AC5+ATP. Global movement obtained by PCA on the MD simulation of AC5+ATP (mode 2, note that the first two modes have very similar eigenvalues in this case). The beta-sheet β2 is no longer mobile. The opening of the angle between the helices α1 and α2 is coupled with the closing of the angle between the helices α3 and α4 and with the closing of interface between the two domains. Loops in domain C2, at either end of helix α3, also undergo coupled movements.
(MOV)

S3 Video PCA on the MD simulation of AC5+Gsα. Global movement obtained by PCA on the MD simulation of AC5+Gsα (mode 1). The translation of helix α2 along its axis is coupled with the change of position of the beta-sheet β2. The movement of helix α2 also opens the angle with helix α1 and this is coupled with closing of the angle between the helices α3 and α4 and the relative rotation of the two domains, opening the domain interface towards the viewer and closing it on the far side.
(MOV)

S4 Video PCA on the MD simulation of AC5+ATP+Gsα. Global movement obtained by PCA on the MD simulation of AC5: ATP+Gsα (mode 1). The beta-sheet β2 is highly mobile. Its movement is mainly coupled with intra-domain movements, although some changes occur in helix α3 and with the loops at the closed end of helices α3 and α4.
(MOV)
