# Supplementary material for: A molecular dynamics study of adenylyl cyclase: The impact of ATP and G-protein binding
Source: PLoS One. 2018 Apr 25;13(4):e0196207. doi: 10.1371/journal.pone.0196207 (PMC5918993; doi:10.1371/journal.pone.0196207)
Supplement: S1 Table — To compute the RMSD we used the average structure as the reference state. (PDF) [file pone.0196207.s001.pdf]

S1 Table. Average and standard deviation of RMSD for the C1 and C2 domains of AC5, α-helix angles for both domains and characterization of the C1/C2 interface: gap volume, change in accessible surface area (ΔASA) and gap index, for the systems studied with molecular dynamics simulation. To compute the RMSD we used the average structure as the reference state.

**Data**	**AC5**	**AC5+ATP**	**AC5+ATP+Gsα**	**AC5+Gsα**
RMSD C1 (Å)	1.0 ± 0.2	0.9 ± 0.2	0.8 ± 0.1	1.0 ± 0.2
RMSD C2 (Å)	1.2 ± 0.4	1.2 ± 0.2	1.1 ± 0.2	0.9 ± 0.2
α_{C1} (°)	26 ± 4	44 ± 6	42 ± 5	31 ± 6
α_{C2} (°)	50 ± 4	43 ± 5	47 ± 3	43 ± 4
Gap Volume ($Å^3$)	7021 ± 544	6183 ± 418	7319 ± 542	6872 ± 479
ΔASA ($Å^2$)	2311 ± 161	2258 ± 96	1927 ±1 43	2236 ± 91
Gap index (Å)	3.1 ± 0.3	2.8 ± 0.3	3.8 ± 0.5	3.1 ± 0.3
