# Supplementary material for: A molecular dynamics study of adenylyl cyclase: The impact of ATP and G-protein binding
Source: PLoS One. 2018 Apr 25;13(4):e0196207. doi: 10.1371/journal.pone.0196207 (PMC5918993; doi:10.1371/journal.pone.0196207)
Supplement: S3 Table — (PDF) [file pone.0196207.s003.pdf]

S3 Table. Average and standard deviation of RMSD, ASA and ΔASA with domains C1 and C2 for ATP bound to AC5 in the absence or in the presence of Gsα and in the presence of FOK.

**Data**	**AC5+ATP**	**AC5+ATP+Gsα**	**AC5+ATP+FOK**
RMSD ATP (Å)	0.6 ± 0.0	0.3 ± 0.1	0.2 ± 0.1
ASA_{ATP} (Å^2)	94 ± 20	71 ± 14	85 ± 12
$\Delta ASA_{ATP\text{-}C1}$ (Å^2)	263 ± 15	244 ± 13	247 ± 11
$\Delta ASA_{ATP\text{-}C2}$ (Å^2)	259 ± 17	301 ± 19	284 ± 10
