# Supplementary material for: A molecular dynamics study of adenylyl cyclase: The impact of ATP and G-protein binding
Source: PLoS One. 2018 Apr 25;13(4):e0196207. doi: 10.1371/journal.pone.0196207 (PMC5918993; doi:10.1371/journal.pone.0196207)
Supplement: S4 Table — (PDF) [file pone.0196207.s004.pdf]

S4 Table. Average and standard deviation of the gap volume, ΔASA and the gap index of the AC5/Gsα interface in presence and in the absence of ATP.

**Data**	**AC5+ATP+Gsα**	**AC5+Gsα**
Gap Volume ($Å^3$)	3286 ± 333	3424 ± 361
ΔASA ($Å^2$)	1244 ± 123	1067 ± 63
Gap index (Å)	2.7 ± 0.5	3.2 ± 0.4
