# Supplementary figures and images for: A molecular dynamics study of adenylyl cyclase: The impact of ATP and G-protein binding
Source: PLoS One. 2018 Apr 25;13(4):e0196207. doi: 10.1371/journal.pone.0196207 (PMC5918993; doi:10.1371/journal.pone.0196207)

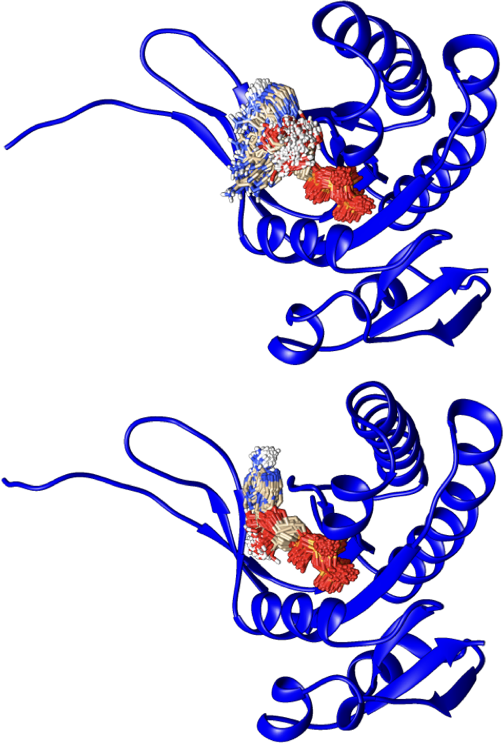

Supplement: S1 Fig — Top: AC5+ATP, Bottom: AC5+ATP+Gsα. In both cases snapshots from the MD trajectories are superposed on the average structure of domain C1 of AC5 (blue). ATP is shown with standard chemical coloring. (TIFF) [file pone.0196207.s005.tiff]

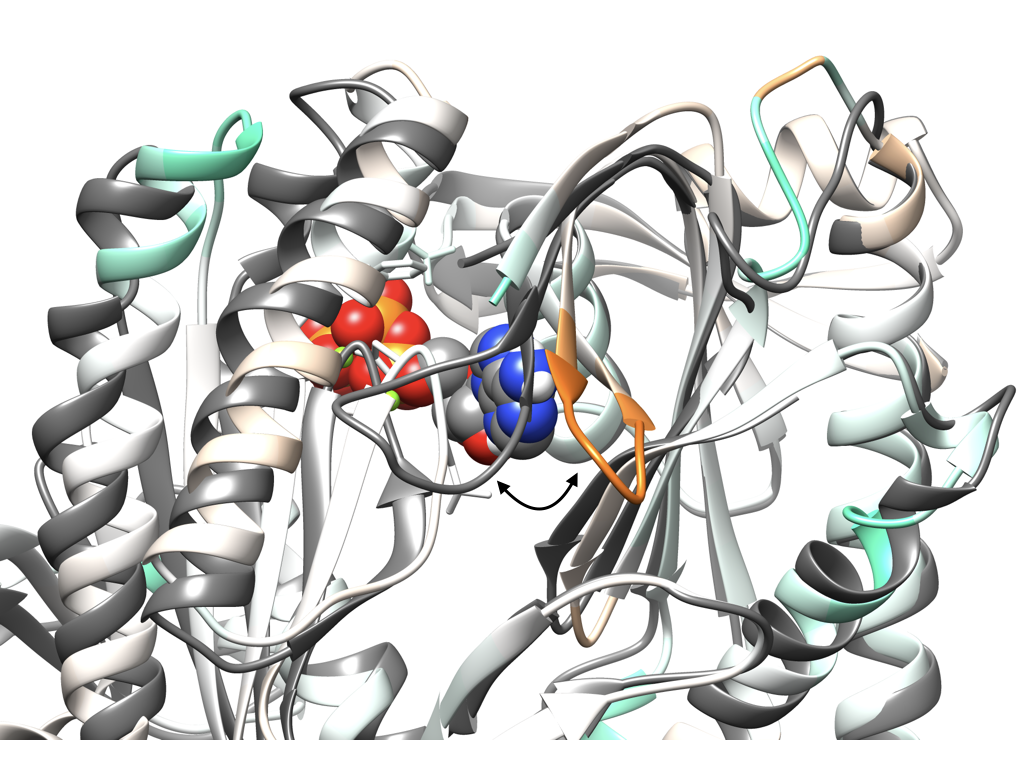

Supplement: S2 Fig — The average structure of the AC5+ATP complex is shown in dark grey (ATP not shown). The same structure after Gsα binding is shown with coloring representing the change in RMSF (the cyan-white-orange variation covering variations of -1.2 Å to + 1.2 Å). (TIFF) [file pone.0196207.s006.tiff]

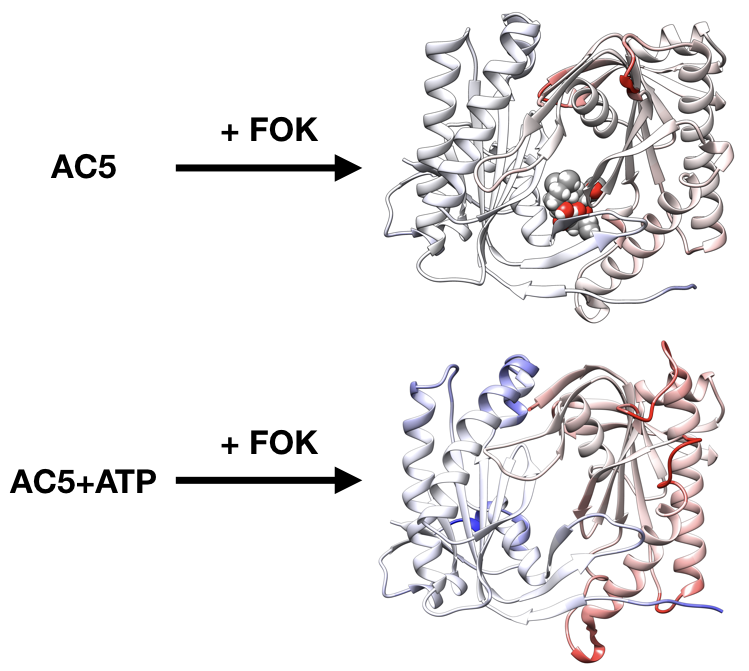

Supplement: S3 Fig — More intense colors (blue for domain C1 and red for domain C2) correspond to larger movements compared to the preceding structure on a scale of 0 → 4 Å. (PNG) [file pone.0196207.s007.png]

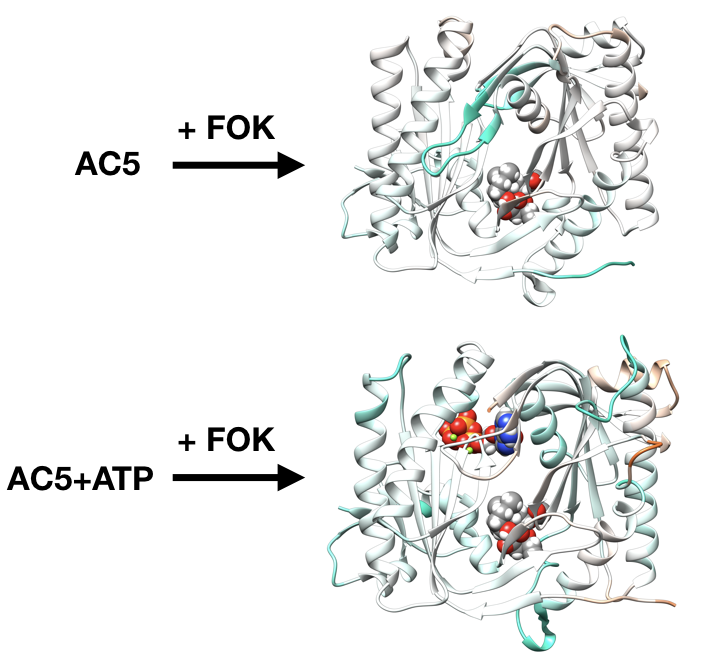

Supplement: S4 Fig — More intense colors (orange for increased flexibility and cyan for decreased flexibility) correspond to differences with respect to the preceding structure on a scale of -1.2 Å → +1.2 Å. (PNG) [file pone.0196207.s008.png]
